# Bladder cancer: a retrospective audit at a single radiation oncology unit of an academic hospital in Johannesburg, South Africa

**DOI:** 10.1186/s43046-024-00241-3

**Published:** 2024-11-04

**Authors:** Trenton Oliver, Duvern Ramiah, Daniel Mmereki, Mia Hugo, Oluwatosin A. Ayeni

**Affiliations:** 1 University of the Witwatersrand, Faculty of Health Sciences, School of Clinical Medicine, Department of Radiation Sciences, Division of Radiation Oncology, University of the Witwatersrand, Faculty of Health Sciences, School of Clinical Medicine, Department of Radiation Sciences, Division of Radiation Oncology, Johannesburg, South Africa; 2 Wits University Donald Gordon Medical Centre, Johannesburg, South Africa

**Keywords:** Bladder cancer in South Africa, Transitional cell carcinoma, Squamous cell carcinoma

## Abstract

**Background:**

Bladder cancer (BCa) is one of the most common urological cancers and remains a leading cause of cancer-related mortality worldwide. Bladder cancer is associated with a range of risk factors, with smoking being one of the most significant contributors. In addition to smoking, exposure to certain chemicals, particularly aromatic amines found in industries such as dye, rubber, leather, and textiles, also increases the risk of bladder cancer. In low-and-middle countries with lower Human Development Index (HDI), data on the underlying causes, incident rate, modes of presentation, treatment, and prognosis of bladder cancer remains unclear and often appear to be inadequate. This study aimed to assess the prevalence, mode of presentation, treatment, and risk factors associated with bladder cancer in Johannesburg, South Africa. By examining these factors, the study seeks to identify possible patterns or predisposing factors that contribute to the development and progression of bladder cancer, which could generate insights that could help to reduce the significant morbidity and mortality associated with this cancer.

**Methods:**

This retrospective study analyzed secondary data from 115 patients who were treated in the radiation oncology unit of an academic hospital between January 2010 and December 2020. By reviewing the medical records of these patients, the study aimed to gather comprehensive information on the prevalence of bladder cancer, modes of presentation, treatment approaches, and associated risk factors. Bladder cancer in this study was assessed using a comprehensive analysis of patient data on demographics, risk factors, clinicopathological aspects, and the specific therapies received. A comparison of patients with squamous cell carcinoma (SCC) and transitional cell carcinoma (TCC) of the bladder was conducted as part of this study. This comparison aimed to explore differences in demographic profiles, risk factors, clinicopathological characteristics, and treatment outcomes between these two histological subtypes.

**Results:**

A total of 115 patients presenting with bladder cancer symptoms were referred to the academic hospital for evaluation and treatment. The incidence rate of bladder cancer was highest among patients with a mean age of 60.7 ± 14.9. Males constituted 60.9% of the cases, resulting in a male-to-female ratio of 1.6:1. The most common risk factors associated with bladder cancer complications included smoking, being male, black ethnicity and increasing age. Transitional cell carcinoma remained the most prevalent histological subtype at the academic hospital, compared to squamous cell carcinoma (SCC). Patients with transitional cell carcinoma (TCC) were more likely to be older (odds ratio (OR): 1.03, 95% Confidence Interval (CI): 1.01–1.06, *p* = 0.029), male (OR: 2.60, 95% CI:1.10–6.04, *p* = 0.030). The study also found that most of the TCC cases were among black patients, though white patients were four times more likely to present with TCC compared to SCC (OR:4.22, 95% CI: 1.43–12.48, *p* = 0.009).

**Conclusion:**

Bladder cancer is still widespread in LMICs, with lower HDI, with elderly males being at risk. To aggressively prevent mortality and morbidity from bladder cancer, bladder cancer health awareness must be maintained to improving prevention, as well as early detection, management and comprehensive patient care and health services for bladder cancer patients. These findings highlight the importance of targeted screening and prevention strategies for high-risk groups, particularly older males with a history of smoking.

## Background

Bladder cancer (BCa) is a prevalent urological cancer, [2], accounting for 3% of all cancer diagnoses globally. It is particularly prevalent in high HDI countries [21], with the highest incidence rates observed in men in Southern and Western Europe, North America, Northern Africa and Western Asia [10]. It is also stated that it is still a concern in low HDI countries, and its prevalence is increasing. Bladder cancer is the tenth most common cancer in the world (with 573 278 new cases per year) and the thirteenth in terms of mortality rate, with 212 536 deaths in 2020 [2, 21]. Radiation treatment is critical for successful therapy, particularly with the increasing incidence of bladder cancer. Comprehending and applying advanced methods is crucial for achieving optimal treatment outcomes, especially in nations undergoing rapid economic development and change.

Several studies have explored development of cancer because of therapy. A study conducted in Europe and Canada by Kaldor et al., [13] found an increased likelihood of bladder cancers in women who had undergone treatment for ovarian cancer, particularly those who received both radiation therapy (RT) and chemotherapy, with a relative risk (RR) of 5.2, 95% CI: 1.6–16 and for those who received RT alone, RR of 1.9,95% CI: 0.77–4.9 compared to patients who received surgery alone [13]. Bladder cancer is frequently overlooked [18], with a notably high-risk following radiation for pelvic tumours (RR 1.75, 95% Cl: 1.50–2.05) [6]. Tobacco smoking and exposure to chemicals are both considered risk factors. Bladder cancer is more common in men, especially among African ethnicities [3]. Limited studies in South African indicated differences in bladder cancer rates based on ethnicity [5, 8, 20]., with coloured men having the highest number of cases [20]. Enhanced diagnostic methods and greater health education are essential for inequalities in BCa treatment [19]. Having knowledge of risk factors and treatments is crucial for patient care especially in LMICs, with low HDI. More investigation is necessary to develop tailored risk-reduction strategies for preventing and treating BCa and reforming public health policies.

This study aimed to assess the prevalence, mode of presentation, treatment, and risk factors associated with BCa a radiation unit of a Johannesburg Academic Hospital, South African. The goal was to identify possible patterns and predisposing factors that could reduce the significant morbidity and mortality associated with this cancer. By analysing patient demographics, clinical presentations, treatment approaches, and risk factors, the study sought to generate insights that could inform public health strategies, enhance early detection, and optimize treatment protocols, ultimately improving outcomes for bladder cancer patients in this region.

## Methods

### Study design and participants

A retrospective study was conducted on all histologically diagnosed cases of BCa referred to the urological cancer outpatient in a radiation oncology unit at a single South African academic hospital. The study period spanned from January 2010 through December 2020. By reviewing the medical records of these patients, the study aimed to analyse various aspects of bladder cancer, including prevalence, mode of presentation, risk factors, and treatment modalities, to identify potential trends that could improve clinical outcomes and reduce the burden of this disease in the region. Patients with a histological diagnosis of bladder cancer, who were referred to the radiation oncology unit for treatment between January 2010 and December 2020, and who were 18 years or older with a registered assessment consultation case record, were included in this study. Exclusion criteria comprised patients who did not have histopathological confirmation of bladder cancer, as well as those whose medical records were missing or could not be retrieved. These criteria ensured that only verified cases with complete and accessible medical histories were included in the analysis, providing reliable data for the study's objectives.

Assessed patient characteristics included demographics, identifiable risk factors, and clinicopathological features. Demographics data of the patients encompassed age, gender, race, along with histological, radiological findings, and outcomes. The histological data involved detailed analysis of tumor type, such as transitional cell carcinoma (TCC) or squamous cell carcinoma (SCC), while radiological findings provided insights into tumor size, location, and the extent of disease spread. Smoking, schistosomiasis, a family history of bladder cancer, a history of urolithiasis (kidney stones), previous pelvic radiotherapy, and prior chemotherapy were all identifiable risk factors for bladder cancer in this study. Clinical characteristics, including comorbidities and performance status, were recorded for each patient. Performance status often evaluated using scales like the Eastern Cooperative Oncology Group (ECOG), was also recorded to assess the patient's overall physical functioning and ability to tolerate various treatment modalities at time of presentation. The following scores were assigned to evaluate patient functionality: 0 = asymptomatic, 1 = restricted in physically strenuous activity but ambulatory and able to carry out light or sedentary work, 2 = unable to work but out of bed for more than 50% of waking hours, 3 = limited self-care; confined to bed or chair for more than 50% of waking hours, 4 = no self-care, completely disabled, and completely confined to bed or chair. These scores helped assess each patient's general health status and ability to tolerate and respond to bladder cancer treatments.

Pathological features reported in radical cystectomy tissues included the histopathological variant of bladder tumor. The variants analyzed included: TCC, SCC and others atypical, mixed variant or adenocarcinoma. The stage of disease was recorded for each patient and categorised as follows: Non-muscle Invasive bladder cancer (NMIBC), muscle-invasive bladder Cancer (MIBC), or metastatic urothelial cancer (MUC). These classifications helped guide treatment strategies and provided a clearer understanding of patient prognosis based on the extent of disease progression. Therapy-related variables identified in the study included the treatment modalities such chemotherapy (intravesical, neoadjuvant or adjuvant), surgery (transurethral resection of bladder tumour, radical cystectomy, or urinary diversion), and radiation therapy (radical or palliative). These therapy-related variables were crucial in analysing the different treatment approaches and their impact on patient outcomes. The assessment included looking at the radiation dosage received/fractionation (conventional versus hypofractionation timeline), and the concomitant chemotherapy and radiotherapy (chemoradiation) were evaluated. There were also documented different reasons for palliative radiotherapy, treatment outcomes, and toxicities.

The Chief Executive Officer (CEO) of a South African academic hospital granted permission to access patients’ medical records. The retrospective study was approved by the Human Research Ethics Committee (HREC) of the University of the Witwatersrand, and the need for informed consent was waived (Certificate Number: M211024). Prior to conducting analysis, the patient data was anonymized and de-identified.

### Statistical analysis

Categorical variables were tabulated using both absolute frequencies and proportions. Mean and standard deviation (SD) were used to report continuous variables after assessing for normality with histogram plot super-imposed with a standardized curved. A Shapiro Wilk test was performed to determine the normality of the variables. A bivariate logistic regression between variables was done using the Chi-square test and logistic regression was conducted to examine the relationships with the histopathological variants. The odds ratio (OR) with a 95% confidence interval (CI) and *p*-values were tabulated. Throughout the analysis, variables were considered significant if their *p*-values was < 0.5. All analyses were performed using Stata version 16 (StataCorp LP, College Station, TX, USA).

## Results

### Demographic profiles

Between 2010 and 2020, 115 BCa patients were referred to the academic hospital’s radiation oncology unit, a majority of 115 BCa patients were male, comprising 60.9% (*n* = 70) of the total. Importantly, the study found that 52.2% (*n* = 60) of the male patients did not have a history of smoking. When stratifying the cases by race, the incidence was highest among the African race (52.2%, *n* = 60), followed by white patients (*n* = 36, 31.3%), coloured (*n* = 13, 11.3%), and Asians (*n* = 6, 5.2%). In Table 1, the age standard deviation is 60.7 ± 14.9 years, with 2.6% (*n* = 3) of patients having chemotherapy, and 1.7% (*n* = 2) having pelvic radiotherapy. The study utilized individual history to analyse prior pelvic radiation received; but the association with second primary cancer was challenging due to a lack of available information on secondary causes of bladder cancer. The study found that family history of bladder cancer, 6.1% (*n* = 7), history of schistosomiasis, less than 5% (*n* = 5), and urolithiasis, 0.9% (*n* = 1) were less frequently indicated in this group. When the 115 cases were analyzed, it was observed that many of the patients had an ECOG performance status of 2 (40.0%) and 1 (36.5%), respectively.

**Table 1:** Socio-demographic characteristics of patients diagnosed with bladder cancer from 2010 to 2020

**Variables**	**Categories**	**Frequencies *N*=115**	**Percentages** **(%)**
Age in years, mean ± SD	60.7 ± 14.9		
Sex	Male	70	60.9
	Female	45	39.1
Sex ratio	Male: Female	1.6:1	-
Race	African	60	52.2
	White	36	31.3
	Coloured	13	11.3
	Indian	6	5.2
History of smoking	No	60	52.2
	Yes	55	47.8
History of schistosomiasis	No	110	95.7
	Yes	5	4.3
History of Urolithiasis	No	114	99.1
	Yes	1	0.9
Previous pelvic radiotherapy	No	113	98.3
	Yes	2	1.7
Previous chemotherapy	No	112	97.4
	Yes	3	2.6
Family history of bladder cancer	No	108	93.9
	Yes	7	6.1
^a^ECOG performance status	0	7	6.1
	1	42	36.5
	2	46	40.0
	3	18	15.7
	4	2	1.7

^a^ECOG (Eastern Cooperative Oncology Group)

The functional status of the patients at presentation was also recorded. The scores were categorised as follows: 0 = asymptomatic, 1 = restricted in physically strenuous activity, 2 = unable to work but out of bed for more than 50% of waking hours, 3 = limited self-care, confined to bed or chair more than 50% of waking hours, 4 = no self-care, completely disabled, totally confined to bed or chair. Many of the patients had a functional status score of 2 (*n* = 46; 40.0%) and 1 (*n* = 1, 36.5%).

In terms of comorbidities of the patients analyzed, 33.0% (*n* = 38) had hypertension, 24.3% (*n* = 28) had chronic kidney disease, 12.2% (*n* = 14) had diabetes, 7%(*n* = 8) were HIV positive, and 5.2% (*n* = 6) had thromboembolic disease (VTD). The study found that 5.2% (*n* = 6) of the patients were diagnosed with Veno-thromboembolic disease (VTE). There were no indications of arthritis or autoimmune conditions in the patients’ medical history.

### Histological characteristics of the disease

**Fig. 1:**
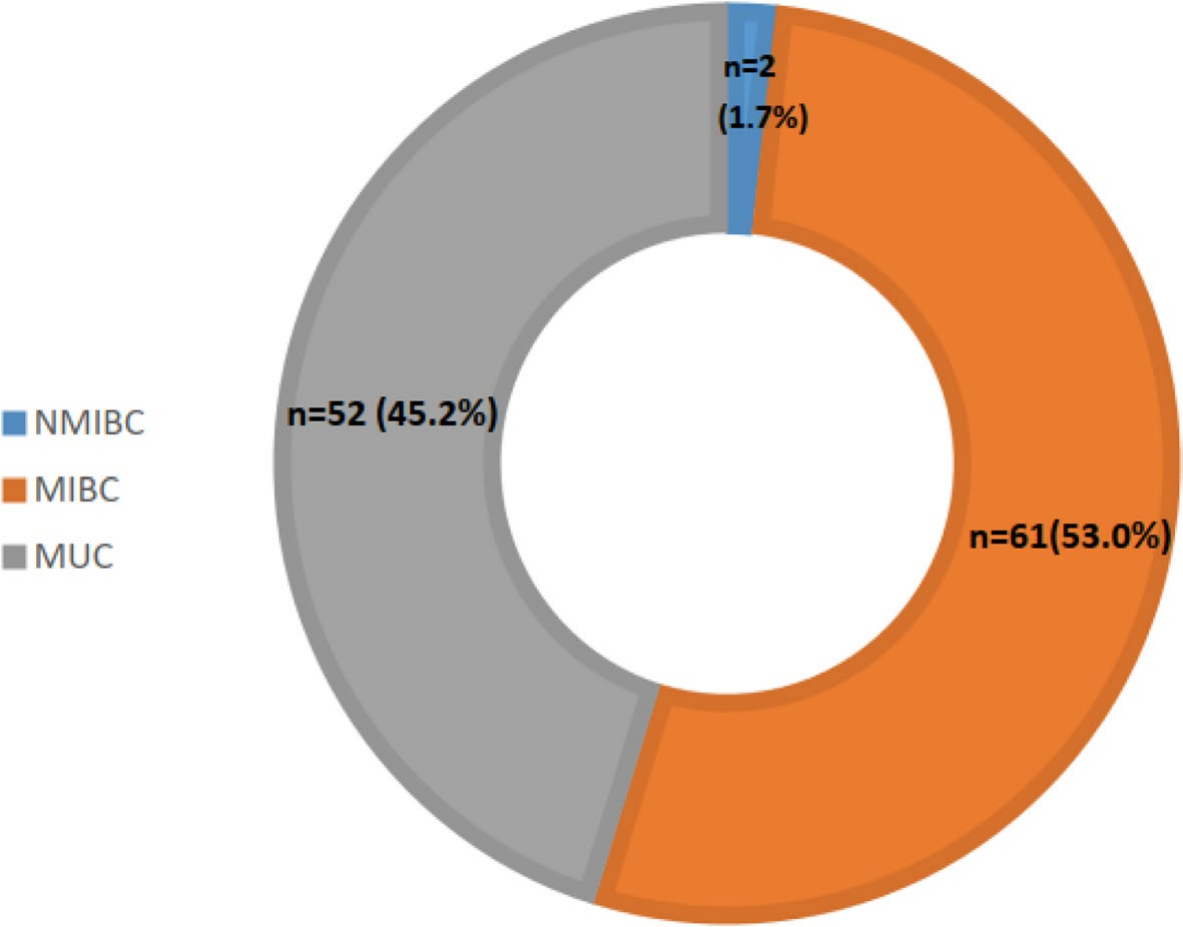
Distribution of stage at presentation of patients diagnosed with bladder cancer *NMIBC Non-Muscle Invasive Bladder Cancer, MIBC Muscle-Invasive Bladder Cancer, MUC Metastatic Urothelial Cancer

In the analysis of clinicopathologic features, it was found that 53.0% (*n* = 61) of the patients had Muscle-Invasive Bladder Cancer (MIBC), 45.0% (*n* = 52) had Metastatic Urothelial Cancer (MUC), and the remaining 2.0% (*n* = 2) had Non-Muscle Invasive Bladder Cancer (NMIBC). This distribution highlights the advanced stage of disease at diagnosis for most patients in the study, with a large proportion presenting with either muscle-invasive or metastatic disease, emphasizing the need for improved early detection and treatment strategies (Fig. 1).

The pathological presentation of symptoms for patients with BCa is detailed in Table 2. When examining at the histological characteristics of bladder cancer in the cohort diagnosed at the academic hospital between 2010 and 2020, it was observed that majority of the patient (67.8%, *n* = 78) presented TCC, while (27%, *n* = 31) were diagnosed with SCC (See Table 2). This finding aligns with the global trend where TCC is the most common histological subtype of bladder cancer, emphasizing the need to focus on this variant in terms of diagnosis, treatment, and research efforts. Among the BCa patients in this cohort, 80.8% (*n* = 93) presented with grade three disease, indicating high prevalence of aggressive, poorly differentiated tumours. In contrast, grades one and two accounted for 5.2% (*n* = 6) and 13.9% (*n* = 16) of the patients, respectively. In terms pathological classification, 29.6% (*n* = 34) of BCa patients exhibited lymphvascular space invasion (LVSI). LVSI is a critical pathological feature that indicates the presence of cancer cells within the lymphatic or blood vessels.

**Table 2:** The pathological characteristics of bladder cancer diagnosed at the academic hospital between 2010 and 2020

Variables	Categories	Frequencies	Percentages
Histology	TCC	78	67.8
	SCC	31	27.0
	Atypical	6	5.2
Grading	3	93	80.9
	2	16	13.9
	1	6	5.2
LVSI	No	81	70.4
	Yes	34	29.6

an *SCC* Squamous cell carcinoma, *TCC* Transitional cell carcinoma, *LVSI* lymphovascular space invasion

### Characteristics of bladder *cancer* patients' treatment

Different bladder cancer-related therapies were initiated at the time diagnosis for all patients referred to the academic hospital’s division of radiation oncology, as detailed in Table 3. These therapies included chemotherapy, surgery and radiation therapy. In this cohort, 3.5% (*n* = 4) of the patients had previously received intravenous chemotherapy, while 5.2% (*n* = 6) had undergone platinum-based neoadjuvant chemotherapy, specifically with cisplatin and gemcitabine. In this cohort, Transurethral resection of bladder tumour (TURBT) was performed in 67.0% (*n* = 77) while radical cystectomy was carried out on 21 patients (18.3%).

**Table 3:** Bladder cancer-related therapy between 2010 and 2020

Group	Variables	Categories	Frequencies *N* = 115	Percentages (%)
Previous chemotherapy	Intravesical	No Yes	111 4	96.5 3.5
	Neoadjuvant	No Yes	109 6	94.8 5.2
	Adjuvant	No(No patients had previous adjuvant chemotherapy)	115	100.0
Previous surgery	TURBT	Yes No	77 38	67.0 33.0
	Cystectomy	No Yes	94 21	81.7 18.3
	Urinary diversion	No Yes	69 46	60.0 40.0
Previous bladder radiation therapy	CCRT	No Yes	114 1	99.1 0.9
	RT Alone	No (No patients had previous RT alone)	115	100.0
Treatment Planned		Palliative Radical No treatment planned	68 26 21	59.1 22.6 18.3

an *TURBT* Transurethral resection of bladder tumour, *CCRT* Concurrent chemoradiotherapy, *RT* Radiation therapy

Among the 21 (18.3%) of the patients who underwent cystectomy, post-surgery outcomes revealed that 52.4% (*n* = 11) had LVSI, and 47.6% (*n* = 10) exhibited Perineural invasion (PNI). Positive margins (defined as a tumour on ink) and perineural invasion (PNI) were the primary causes of pelvic recurrence in 47.6% (*n* = 10/21) of the patients before adjuvant radiotherapy, while node-positive disease (defined as lymph nodes detected in the pelvic dissection) accounted for 28.6% (*n* = 6/21) of the patients at high risk of pelvic recurrence following cystectomy. 9.5% (*n* = 2) of the 21 patients with incomplete resection, defined as a subtotal resection with gross residual disease, were referred for radiation.

It is standard practice for us to initiate a treatment plan (i.e., develop a roadmap that helps to set out the expected path of treatment, including determining the exact dose and duration of the treatment). The cancer care team develops this and provides it to the patient and anyone else who may require information about the intended course of therapy for bladder cancer patients [17]. The study found that 81.7% (*n* = 94) of the 115 BCa patients were planned for radiation therapy on average. Palliative care was prescribed to 59.1% (*n* = 68) of patients, radical treatment to 22.6% (*n* = 26), and no therapy was recommended to 18.3% (*n* = 21) of patients who presented to the division for various reasons.

**Fig. 2:**
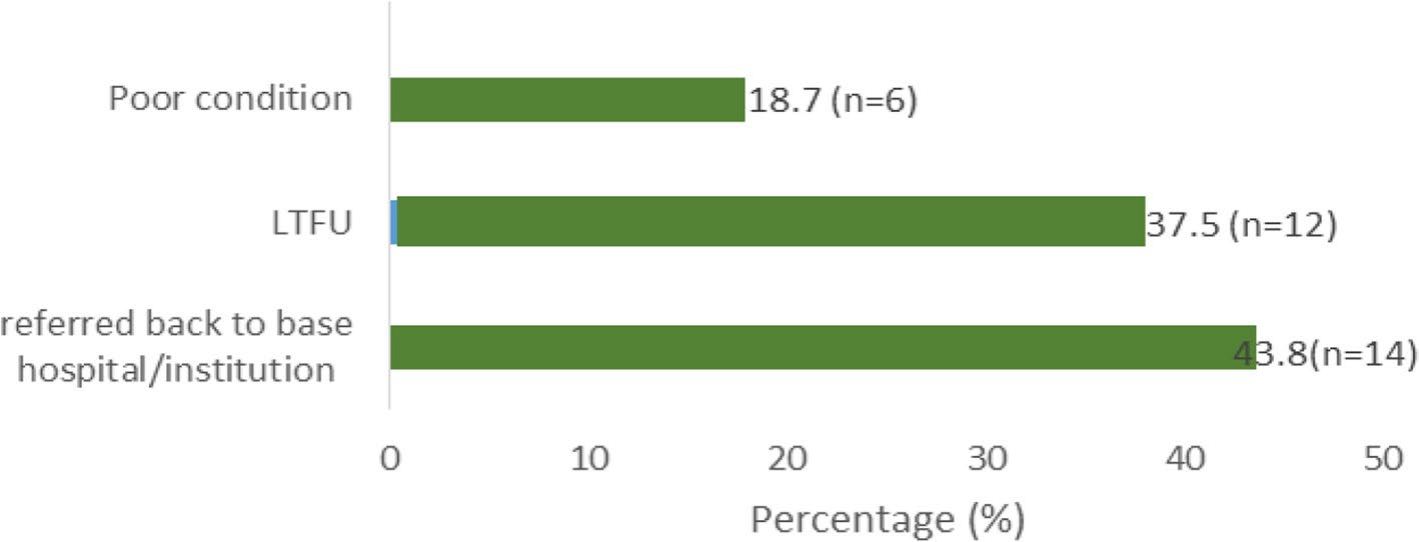
Reasons for non-completion of radiotherapy in patients diagnosed with bladder cancer between 2010 and 2020. *LFTU Lost to follow-up

Of the 94 (81.7%) patients who were scheduled to get radiation therapy, only two-thirds (66%, *n* = 62) completed their treatment, while the remaining 32 (34.0%) patients did not (Table 4). Figure 2 illustrates the reasons not to complete radiation. Among the reasons for non-completion of radiotherapy were referrals to their base hospital or supportive treatment for 37.5% (*n* = 12/32), lost to follow-up (LTFU) for 18.7% (*n* = 6/32), and poor conditions for 18.7% (*n* = 6/32). In this cohort, 38.2% (*n* = 26) of the 68 patients received referrals for palliative care, while 23.6% (*n* = 16) received recommendations for chemotherapy. Out of the 68 patients, 22 (32.4%) were lost to follow-up, 2 (2.9%) discontinued treatment, and two (2.9%) died. Similarly, 88.5% (*n* = 23/26) of the group that received a high dosage of radiation, were lost follow-up (LFTU). In contrast, only two patients (7.7%) received follow-up surveillance following treatment, and one (3.8%) received a referral for palliative chemotherapy.

**Table 4:** Treatment planned and outcome categories in patients diagnosed with bladder cancer between 2010 and 2020

**Outcome categories**				
	**Palliative Treatment** ***n* = 68(59.1%)**	**Radical treatment** ***n* = 26(22.6%)**	**No treatment planned** ***n* = 21(18.3%)**	**Total** ***n* = 115(%)**
Lost to follow-up	22(32.4)	23(88.5)	11(52.4)	56(48.7)
Referred for palliative care	26(38.2)	0	1(4.7)	27(23.5)
Referred for palliative chemotherapy	16(23.6)	1(3.8)	6(28.6)	23(20.0)
Referred for surgery	0	0	3(14.3)	3(2.7)
Discontinuation of treatment due to poor condition	2(2.9)	0	0	2(1.7)
Demised	2(2.9)	0	0	2(1.7)
Under follow-up for surveillance	0	2(7.7)	0	2(1.7)

Among the prevalent symptoms recorded for palliative radiotherapy were “inoperable disease” (*n* = 29/68 (42.7%)), followed by "bleeding from a tumour" (*n* = 14/68 (20.6%)) (Table 5). The data reported that one patient (1.5%) was diagnosed with a bulky and symptomatic lung metastasis, requiring palliative radiotherapy.

**Table 5:** Indications for palliative radiotherapy (*n* = 68)—(59.1%) in patients diagnosed with bladder cancer between 2010 and 2020

Group name	Frequencies	Percentage
Inoperable disease	29	42.6
Bleeding	14	20.6
Intolerable Pelvic Pain	10	14.7
Spinal Cord Compression	6	8.8
Bone Metastasis	5	7.4
Brain Metastasis	3	4.4
**Symptomatic** Bulky Lung Metastasis	1	1.5

Table 6 presents the dose fractionation and total dosage of radiation doses administered to the patients. The table outlines the specific fractionation regimens used, detailing the number of fractions and the total cumulative radiation dosage delivered. These schedules vary based on the individual treatment goals, such as curative intent versus palliative care. Most the patients received a palliative fractionation schedule (< 30 Gy) of radiotherapy (72.3%, *n* = 68), which is commonly used to relieve symptoms and improve quality of life in patients with advanced or metastatic bladder cancer. During their planned radical treatment, the 26 patients received various fractionation schedules: 20 (21.3%) received a hypofractionated dose (40-55 Gy), while 6 (6.4%) patients received a conventional dose. Only two patients (2.1%) received concurrent chemotherapy platinum-based regime (Cisplatin) with radiation treatment.

**Table 6:** Details of treatment prescribed for patients diagnosed with bladder cancer between 2010 and 2020

Group	Variables	Categories	Frequencies *n* = 94	Percentages (%)
Fractionation (Dose)	Palliative	< 30 Gy	68	72.3
	Hypofractionation	40–55 Gy	20	21.3
	Conventional	60–66 Gy	6	6.4
Concurrent	Cisplatin	Yes	2	2.1
Chemotherapy		No	92	97.9
Completed treatment		Yes	62	66.0
		No	32	34.0

As shown in Table 7, various treatment-related side effects and toxicities were observed among the patients undergoing radiation therapy for BCa. Acute toxicities, defined as adverse effects occurring during therapy and lasting up to three months post-treatment [9], were experienced by many of the patients: more than three-quarters, 86,2% (*n* = 81), of patients experienced early side effects during or shortly after their treatment. Less than one-third (*n* = 26, 27.7%) of the patients reported fatigue as a treatment-related side effect. Genitourinary toxicities were also documented: 2.1% (*n* = 2) experienced G1-cystitis, 5.3% (*n* = 5) experienced G2-cystitis, and only 1.1% (*n* = 1) experienced G3 cystitis. Gastrointestinal toxicities were noted as early side effects: 14.9% (*n* = 14) had Garade 1, 6.4% (*n* = 6) had Grade 2 and 4.3% (*n* = 4) had Grade 3. Radiation dermatitis was also a concern, 8.5% (*n* = 8) and 12.8% (*n* = 12) experienced Grade 1 and 2 acute radiation dermatitis, respectively. Leukopenia was less common, affecting only 3.2% of cases. Importantly, there were no recorded deaths during radiation therapy. However, 9.6% (*n* = 9) of the patients did not complete treatment due to adverse radiation therapy side effects and were subsequently lost to follow-up. The median follow-up time for the cohort was 48 days, with an interquartile range (IQR) of 7–99 days, indicating variability in the duration of follow-up among patients.

**Table 7:** Early, late and follow-up treatment outcomes of patients diagnosed with bladder cancer between 2010 and 2020

Group n(%)	Variables n(%)	Categories	Frequencies (n)	Percentages (%)
Early side effects 81(86.2)	Fatigue 26(27.7)	Yes	26	27.7
	Gastrointestinal 24(25.5%)	Grade 1 Grade 2 Grade 3	14 6 4	14.9 6.4 4.2
	Skin 20(21.3)	Grade 1 Grade 2	8 12	8.5 12.8
	Genitourinary 8(8.5)	G1-cystitis G2-cystitis G3-cystitis	2 5 1	2.1 5.3 1.1
	Haematological 3(3.2)	Leukopenia	3	3.2
Follow-up	Lost to follow up before completing treatment	No Yes	85 9	90.4 9.6

*G* Grade

When it comes to sex-related bladder cancer analysis, smokers were significantly more likely to be men (76.4% vs 46.7%, *p* = 0.001), indicating statistically significant association between smoking and male gender. However, here was no statistically significant relationship between treatment received and smoking status, implying that smoking did not influence the choice or type of treatment administered to patients in this cohort (Table 8).

**Table 8:** Association of gender and treatment received with the smoking status of patients presenting with bladder cancer between 2010 and 2020

Variables	Non-smoker	Smoker	*P*-value
**Gender, *N* = 115 (%)**			
Male	28 (46.7)	42 (76.4)	**0.001**
Female	32 (53.3)	13 (23.6)	
Treatment received, *n* = 94 (%)			
Radical	11 (22.9)	10 (24.4)	**0.870**
Palliative	**37 (77.1)**	**31 (75.6)**	

When comparing patients who with TCC, and SCC, several key differences emerged: patients with TCC were significantly older than those with SCC (62.5 vs 55.0 years, *p* = 0.029). The analysis showed that for every one-year increase in age, patients were 3% more likely to present with TCC. When factors associated with TCC and SCC diagnostic subtypes were analysed, TCC was more likely to be white patients (37.2% vs 16.1%) and in males (67.9% vs 45.2%) compared to SCC patients (Table 9). Transitional cell carcinoma was four times more common in white patients than in black patients (OR:4.22, 95% CI: 1.43–12.48, *p* = 0.009). Additionally, males were more than twice as likely as females to have TCC (OR: 2.60, 95% CI:1.10– 6.04, *p* = 0.030). Although there was no statistically significant association between cigarette smoking and TCC overall, amongst smokers, white patients were six times more likely to have TCC (OR: 6.00, 95% CI 1.32– 27.19, *p* = 0.022). This indicates a strong racial disparity in the impact of smoking on TCC diagnosis.

**Table 9:** Bivariate analysis of factors associated with TCC and SCC diagnosed subtypes

Variable	SCC	TCC	Bivariate analysis	*P*-value
	*N* = 31 (%)	*N* = 78 (%)	OR (95% CI)	
Age in years, mean ± SD	55.0 ± 17.2	62.5 ± 13.8	1.03 (1.01–1.06)	**0.029**
Race				
African	24 (77.4)	33 (42.3)	1.00 (Reference)	
Coloured	2 (6.5)	10 (12.8)	3.64 (0.73–18.13)	**0.115**
Indian/Asian	0 (0.0)	6 (7.7)	Undefined	
White	5 (16.1)	29 (37.2)	4.22 (1.43–12.48)	**0.009**
Gender				
Female	17 (54.8)	25 (32.1)	1.00 (Reference)	**0.030**
Male	14 (45.2)	53 (67.9)	2.60 (1.10–6.04)	
Smoking status				
Non-smoker	17 (54.8)	39 (50.0)	1.00 (Reference)	
Smoker	14 (45.2)	39 (50.0)	1.20 (0.53–2.79)	**0.649**
The race for smokers				
African	9 (64.3)	10 (27.0)	1.00 (Reference)	
Coloured	2 14.3)	5 (13.5)	2.25 (0.35–14.61)	**0.396**
Indian/Asian	0 (0.0)	2 (5.4)	Undefined	
White	3 (21.4)	20 (54.1)	6.00 (1.32–27.19)	**0.022**
Stage				
1	17 (54.8)	41 (54.0)	1.00 (Reference)	
2	14 (45.2)	35 (46.0)	1.04 (0.45–2.39)	**0.933**

*SD* Standard deviation, *SCC* Squamous cell carcinoma, *TCC* Transitional cell carcinoma, *OR Odds ratio*, *CI* Confidence interval

## Discussion

This study assessed the prevalence, mode of presentation, treatment and risk factors associated with bladder cancer in 115 patients referred to a South African academic hospital over an eleven-year period (2010 and 2020). The aim was to identify potential patterns or predisposing factors that could help reduce the significant morbidity and mortality associated with this cancer. This study provided the basis for analysing the prevalence, risk factors, treatment modalities, and outcomes of bladder cancer within this healthcare setting, with a particular focus on identifying any trends that could improve patient management and reduce mortality rates associated with bladder cancer. The reported data clearly show that the mean age of 60.7 ± 14.9 years, suggesting that bladder cancer is associated to aging. The most common risk factors associated with bladder cancer complications included smoking, male gender, Black ethnicity, and increasing age. This is consistent with the findings of several studies, such as Kamal et al. [14] and Koti et al. [15], which reported a median age at diagnosis for urinary bladder cancer of 60 years (range: 40–80 years). These studies highlighted a high incidence and prevalence of cancer of urinary bladder are evident in the sixth decade of life, with a peak occurring in the seventh and eighth, indicating that BCa predominantly affects the elderly population [14]. Similarly, studies from India [12] and Iran [23], found that older age groups had a higher incidence of BCa, with mean ages of 60.2 and 61.9 years, respectively. These consistent findings across regions suggest that aging significantly increases the risk of bladder cancer. Given this association, there is need for enhanced disease management and comprehensive care for elderly bladder cancer. Radiation oncologists and healthcare providers should increase awareness efforts within this high-risk age group, focusing on early detection and prevention strategies. By targeting these older populations, healthcare systems may be able to reduce the morbidity and mortality rates, ultimately improving patient outcomes.

The study found that the overall prevalence of bladder cancer was higher in males (*n* = 70 (60.9%)) than in females. These findings highlight the importance of targeted screening and prevention strategies for high-risk groups, particularly older males with a history of smoking. This aligns with existing literature, where sex is well-established determinant in bladder cancer. Several studies, such as those Sung et al. [21] and Yavari et al. [23], have reported that bladder cancer is significantly more common in men than women. Sung et al. [21] observed that the incidence and mortality rates for bladder cancer are four times those of women globally, with rates of 9.5 and 3.3 per 100,000 men and women, respectively. Burger et al. [4] also supported this finding, attributing the higher incidence in men to the role of male sex hormones in interfering with the body’s ability to fight bladder cancer. Additionally, men are more likely to engage in cancer-predisposing behaviours such as smoking [16]. Sex steroids and their receptors may also play a role in the variable behaviour of TCC between genders, with being a male is associated with higher risk of bladder cancer [14].

Another critical factor is race. Is there a link between race and prevalence of bladder cancer? Race also played a critical role in the prevalence of bladder cancer, consistent with previous research. The study found a significant association between race and bladder cancer, as observed in the Groeneveld et al. [11] study in greater Durban, South Africa, which reported that bladder cancer was about six times more common in Caucasians than in Africans. This could be attributed to the notion that Caucasians are known to have the highest rate of new cancers, and the predominance of black patients with bladder cancer in this study may reflect patterns in healthcare access, where black patients might primarily seek care at this particular hospital, while other races go elsewhere.

The study also highlighted smoking as a significant risk factor for TCC, aligning with literature that estimates smoking accounts for approximately 50–65% of new BCa cases each year. Smoking has been strongly associated with increased risk of TCC (due to exposure to carcinogenic compounds like aromatic amines and N-nitroso compounds found in tobacco, which can cause DNA damage in the form of double-stranded breaks, base modifications, and bulky adduct formation [22, 24]. It has been observed that smoking reflects the socio-demographic characteristics of westernised lifestyles, which are linked to the prevalence of TCC. Westernized lifestyles often include behaviors such as smoking, which is a well-established risk factor for bladder cancer, particularly TCC. A study examining the relationship between smoking and development of bladder cancer found that patients with bladder cancer had a significantly higher smoking index compared to controls. The mean smoking index for BCa was 7.77 ± 3.76 compared to 3.08 ± 1.88 for the control group (*P* < 0.001) [14]. This finding highlights the strong correlation between smoking intensity and bladder cancer risk, reinforcing the need for targeted interventions to reduce smoking as part of bladder cancer prevention strategies. The smoking index, which reflects both the duration and intensity of smoking, serves as an important marker of cancer risk in this population.

A study by Zheng et al. [24] reported that the prevalence of cigarette smoking among men was 77% for TCC cases, 69% for SCC cases, and 65% for controls. Their findings suggested a significant association between cigarette smoking and an increased risk of TCC, with an adjusted odds ratio (OR) of 1.8 (95% confidence interval [CI], 1.4–2.2). Interestingly, smoking was not significantly associated with SCC in their study. Furthermore, the study highlighted those smokers who used cigarettes and pipes had a considerable risk of developing TCC and SCC, with an OR of 2.9 (2.1–3.9) for TCC and 1.8 (1.2–2.6) for SCC. These findings underscore the strong link between smoking and the risk of bladder cancer, particularly TCC and suggest that combined smoking habits further elevate the risk. While there may be some differences in the exact prevalence rates observed across studies, these data collectively support the hypothesis that smoking history remains a strong risk factor for developing the TCC subtype of bladder cancer. The impact of smoking on SCC appears to be less pronounced, further emphasizing the distinct etiological pathways between these two bladder cancer subtypes.

When comparing histological subtypes, TCC was more prevalent than SCC in this cohort and patients with TCC were more likely to be older, white, and male. White patients were four times more likely to have TCC than black patients, and males were more than twice as likely as females to have TCC. These findings suggest that older age, male gender, and white race are significant factors associated with a higher likelihood of TCC compared to SCC in this cohort. This contrasts with studies in sub-Saharan Africa (SSA), where SCC is more prevalent. TCC is more prevalent in high HDI countries due to higher exposure to carcinogenic chemicals [1]. Groeneveld et al. [11] reported a striking difference in the histological subtypes of BCa between Africa and Caucasian patients. In their study, SCC accounted for 53% of BCa cases in African patients, while TCC constituted 95% of the cancers in Caucasians. This difference likely reflects underlying risk factors unique to each population. In countries like Egypt or other African nations such as Sudan, Kenya, Uganda, Ghana, and Senegal, BCa is predominantly of the squamous cell type. This high prevalence of SCC in these regions is largely attributed to chronic infections, particularly schistosomiasis or bilharziasis [7]. This highlights the critical role that environmental and infectious disease factors play in the epidemiology of bladder cancer in Africa, necessitating targeted public health interventions, such as schistosomiasis control, to reduce the burden of bladder cancer. Another study reported that among the confirmed cases, 689 were diagnosed as SCC, accounting for 35% of cases, 1,197 were TCC (60%). Additionally, 102 (5%) was classified other type of primary bladder cancer cases The study also highlighted the most prevalent comorbidities within the patient cohort, which included hypertension, chronic kidney disease, and diabetes mellitus. These comorbid conditions can complicate the management of bladder cancer, as they may influence both the patient's overall health and their ability to tolerate cancer treatments. The presence of these comorbidities underscores the importance of a holistic approach to patient care, addressing not only the cancer but also the management of these additional health conditions to improve patient outcomes.

Finally, the stage of BCa at diagnosis is crucial of patient outcomes and survival. Early detection and treatment at a less advanced stage can significantly reduce mortality. In our study, the majority presented with advanced stage of the disease, with 53.0% (*n* = 61), having MIBC and 45.0% (*n* = 52) presenting MUC, reflecting late presentation (98%). Only a smaller percentage of the patients (2%) were diagnosed at an early stage, such as NIMBC. Late-stage presentation limits treatment options, often necessitating palliative care rather than curative interventions. Could these patients have been more appropriately assisted at early-stage of the disease? However, in resource-limited settings, where access to early screening and diagnostic services may be constrained, improving early-stage detection is essential. Research has shown that this is not a straightforward issue. A study by Krimphove et al. (2019) reported that female patients with non-muscle invasive bladder cancer (NMIBC) tend to present late with more advanced tumour stages compared to males. Additionally, female patients are more likely to experience early recurrences of the diseases. This delayed presentation in women may be due to several factors, including potential differences in symptom recognition, healthcare-seeking behavior, or biological differences that contribute to more aggressive disease progression in females. However, in the context of Africa, treating late-stage bladder cancer presents a significant challenge, particularly due to limited resources. The scarcity of specialized healthcare services, diagnostic tools, and advanced treatment options in many African countries complicates the management of bladder cancer, especially when patients present with advanced disease stages like Muscle-Invasive Bladder Cancer (MIBC) or metastatic cancer. It is suggested that stage at diagnosis for bladder cancer is critical for providing patients with prompt and effective treatment. Early detection can significantly improve outcomes by enabling timely interventions, such as surgery or localized therapy, before the disease progresses to more advanced stages. Afterwards, care should be kept consistent and continuous, with a focus on maintaining treatment according to established guidelines. To achieve this, healthcare systems should strive for efficiency by streamlining processes and ensuring seamless transitions between different care settings, from initial diagnosis to treatment and follow-up. This requires coordinated efforts across various levels of healthcare, including primary care, specialized oncology services, and post-treatment monitoring. Implementing standard guidelines and protocols can help ensure that patients receive timely, evidence-based care, ultimately reducing bladder cancer morbidity and mortality.

As mentioned above, radiotherapy was the primary treatment modality for BCa in this study, primarily due to its availability compared to other treatment in the region. Patients from district hospitals were often referred to specialists at the academic for radiotherapy. This approach allowed for more comprehensive assessments and facilitated the identification of cases where TURBT and cystectomy of the bladder cancer might be appropriate.

Our indications for palliative treatment in patients with bladder cancer, therefore, include the following situations:

- Inoperable disease
- Bleeding,
- Intolerable Pelvic Pain,
- Spinal Cord Compression,
- Bone Metastasis,
- Brain Metastasis,
- Symptomatic Bulky Lung Metastasis

Treatment of bladder cancer, while necessary, unfortunately still carries significant treatment adverse effects and toxicities. The nature and severity of these side effects vary depending on the type of treatment received, such as surgery, chemotherapy, or radiotherapy. These treatment-related toxicities can significantly impact patients' quality of life and may limit the ability to tolerate aggressive therapies, particularly in elderly or comorbid patients. Ongoing research aims to develop more targeted therapies and supportive care strategies to minimize these adverse effects while maintaining effective cancer control.

As a result of the retrospective study, it is critical to note that some of the possible reasons contributing to the prevalence rate include delayed access to healthcare services, detection, and appropriate treatment in healthcare settings. The higher number of symptoms per patient, the more advanced disease and the more challenging in the natural history prognosis.

One limitation of the present study was the retrospective design, which only included data from a single oncological unit, may limit data’s generalizability. Additionally, the study may contain inherent biases such as incomplete patient-related factor recording and possible exclusion of patients from other departments at the academic hospital. Despite these limitations, the study aimed to highlight information on the epidemiology of bladder cancer, which could potentially be helpful in the prevention of bladder cancer. Furthermore, a lack of multicentre data. A retrospective multicentre study would provide a clearer picture of bladder cancer in South Africa. Another drawback was the lack of information on date of death (missing data on mortality), which potentially hindered the ability to analyse survival outcomes fully. However, the study provides valuable insights into the epidemiology, risk factors, and treatment of BCa in a South African setting. The findings highlight the importance of early detection, targeted awareness efforts, and tailored treatment strategies, particularly for high-risk group such as older male smokers.

## Conclusions

The study's findings indicate the prevalence of bladder cancer in a Johannesburg academic hospital, which is significantly associated with male gender and a smoker, with TCC being more prevalent than SCC. Radiation patients with bladder cancer experience the following adverse effects: fatigue, haematological, genitourinary, gastrointestinal, and skin side effects, which may result in an increase in the burden on South Africa’s public health sector. In conclusion, the data shown in this study emphasise the importance of understanding the etiology of bladder cancer in oncology research in low and middle HDI countries in the context of patient care. Improving the diagnostic and bladder cancer registration practices may assist in reducing demands on health systems, treatment, access, and diagnostic facilities. Enhancing awareness, improving healthcare access, and implementing efficient referral systems can allow for earlier diagnosis, more effective treatment, and ultimately a reduction in bladder cancer mortality. To identify key symptoms of bladder at an early stage, it is important to enhance health awareness and consider oncology’s specific role in patient care. This should also lead to improved patient care and health services. Furthermore, the authors suggest that bladder cancer patient follow-up is crucial step in the trajectory for improving disease data and enhancing tailored treatment better in terms of disease outcome and treatment toxicities, thereby improving the bladder cancer prevention, early detection, and comprehensive patient care and health services.

## Data Availability

All data used in this study are available from the corresponding author.

## Abbreviations

BCa: Bladder cancer
CCRT: Concurrent chemoradiotherapy
CI: Confidence interval
DNA: Deoxyribonucleic acid
ECOG: Eastern Cooperative Oncology Group
G: Grade
HDI: Human development index
HREC: Human Research Ethics Committee
LTFU: Lost to follow-up
LVSI: Lymphvascular space invasion
NMIBC: Non-muscle Invasive bladder cancer
MIBC: Muscle-invasive bladder Cancer or
MUC: Metastatic urothelial cancer
N: Number
OR: Odds ratio
PNI: Perineural invasion
RT: Radiation therapy
RR: Relative risk
SCC: Squamous cell carcinoma
SD: Standard deviation
SSA: Sub-Saharan Africa
TCC: Transitional cell carcinoma
TURBT: Transurethral resection of bladder tumour
TX: Texas
USA: United States of America
VTD: Thromboembolic disease
VTE: Veno-thromboembolic disease
